# Freeze-dried strawberry powder improves lipid profile and lipid peroxidation in women with metabolic syndrome: baseline and post intervention effects

**DOI:** 10.1186/1475-2891-8-43

**Published:** 2009-09-28

**Authors:** Arpita Basu, Marci Wilkinson, Kavitha Penugonda, Brandi Simmons, Nancy M Betts, Timothy J Lyons

**Affiliations:** 1 Department of Nutritional Sciences, 301 Human Environmental Sciences, Oklahoma State University, Stillwater, OK 74078, USA; 2 Harold Hamm Oklahoma Diabetes Center, University of Oklahoma Health Sciences Center, OKC, OK 73104, USA

## Abstract

**Background:**

Strawberry flavonoids are potent antioxidants and anti-inflammatory agents that have been shown to reduce cardiovascular disease risk factors in prospective cohort studies. Effects of strawberry supplementation on metabolic risk factors have not been studied in obese populations. We tested the hypothesis that freeze-dried strawberry powder (FSP) will lower fasting lipids and biomarkers of oxidative stress and inflammation at four weeks compared to baseline. We also tested the tolerability and safety of FSP in subjects with metabolic syndrome. FSP is a concentrated source of polyphenolic flavonoids, fiber and phytosterols.

**Methods:**

Females (n = 16) with 3 features of metabolic syndrome (waist circumference >35 inches, triglycerides > 150 mg/dL, fasting glucose > 100 mg/dL and < 126 mg/dL, HDL <50 mg/dL, or blood pressure >130/85 mm Hg) were enrolled in the study. Subjects consumed two cups of the strawberry drink daily for four weeks. Each cup had 25 g FSP blended in water. Fasting blood draws, anthropometrics, dietary analyses, and blood pressure measurements were done at baseline and 4 weeks. Biomarkers of oxidative stress and inflammation were measured using ELISA techniques. Plasma ellagic acid was measured using HPLC-UV techniques.

**Results:**

Total cholesterol and LDL-cholesterol levels were significantly lower at 4 weeks versus baseline (-5% and -6%, respectively, p < 0.05), as was lipid peroxidation in the form of malondialdehyde and hydroxynonenal (-14%, p < 0.01). Oxidized-LDL showed a decreasing trend at 4 weeks (p = 0.123). No effects were noted on markers of inflammation including C-reactive protein and adiponectin. A significant number of subjects (13/16) showed an increase in plasma ellagic acid at four weeks versus baseline, while no significant differences were noted in dietary intakes at four weeks versus baseline. Thus, short-term supplementation of freeze-dried strawberries appeared to exert hypocholesterolemic effects and decrease lipid peroxidation in women with metabolic syndrome.

## Background

Colorful fruits and vegetables containing a wide range of phytochemicals have been associated with significant cardiovascular health benefits. Strawberries are an especially good source of phytochemicals, particularly anthocyanins and ellagic acid, which have potent antioxidant and anti-inflammatory functions [1]. Apples and strawberries have also been reported to be the largest contributors of cellular antioxidant activity among all fruits consumed in the United States [2]. Strawberry juice extracts have been shown to significantly inhibit free radicals [3], and reduce ox-LDL-induced proliferation of rat aortic smooth muscle cells [4]. Ellagic acid supplementation also reduced oxidative stress and atherosclerotic lesion formation in hyperlipidemic rabbits [5]. da Silva et al., using *in vitro *models, reported the anti-hyperglycemic effects of Brazilian strawberries [6]. In animal models, freeze-dried strawberry powder was shown to reduce obesity and improve glycemic control in mice fed a high-fat diet [7], while mice fed anthocyanin extracts from strawberries demonstrated an up regulation of anti-inflammatory adiponectin gene. [8]. Although there are limited findings related to strawberry supplementation, the data suggest the possibility of strawberries in reducing cardiovascular disease risk factors.

Epidemiological observations also suggest that strawberries may have beneficial cardiovascular effects in women. The Iowa Women's Health Study, a prospective study conducted among 34,489 overweight postmenopausal women, found a significant inverse association between strawberry intake and cardiovascular disease mortality after 16 years of follow-up [9]. The Women's Health study, a cross-sectional study among 26,966 overweight postmenopausal women, further reported that women with higher intakes of strawberries (≥ 2 servings/week) had a borderline significant reduced risk of elevated C-reactive protein (CRP), versus those who consumed none [10]. Thus, these observations suggest the need for additional human studies to determine possible antioxidant, anti-inflammatory, and vascular protective effects of strawberries.

Metabolic syndrome, a significant health problem in the developed countries, is a constellation of risk factors, including atherogenic dyslipidemia (low HDL, high triglyceride), impaired fasting glucose, hypertension, and central adiposity, predisposing to higher risks of oxidative stress, type 2 diabetes and atherosclerotic cardiovascular disease [11-14]. Dietary modifications are one of the key elements in the management of these metabolic abnormalities. Intervention studies using strawberries in humans are limited and have mainly been conducted to investigate the bioavailability and metabolism of strawberry anthocyanins [15,16]. On the basis of this background information, we tested the effects of a freeze-dried strawberry drink on selected cardiovascular risk factors, such as fasting glucose, lipids, and biomarkers of lipid peroxidation and inflammation, as well as safety and tolerability in subjects with metabolic syndrome. Variables of interests were measured at baseline and at the end of the study (4 weeks).

## Methods

### Objectives

To test the hypothesis that freeze-dried strawberry powder supplementation will decrease serum lipids, glucose, oxidative stress and inflammation, we conducted a feeding study among 16 women with metabolic syndrome, consuming 50 g freeze-dried strawberry powder daily for four weeks. In this uncontrolled study, effects on body weight, blood pressure, fasting glucose, lipids, dietary intakes, plasma ellagic acid, biomarkers of oxidative stress and inflammation, safety parameters and tolerance were investigated at baseline and at 4 weeks of the study.

### Study design and subjects

Women (n = 16) ranging in age from 39 to 71 years (mean 51 ± 9.1 years) were recruited at Oklahoma State University (OSU) Stillwater campus during June to October, 2008. All subjects had an elevated BMI (> 30), waist circumference (> 35 inches), elevated systolic blood pressure (> 130 mm Hg), and low HDL(< 50 mg/dL), fulfilling the criteria of metabolic syndrome as defined by the National Cholesterol Education Program, Adult Treatment Panel III [17]. In addition, all subjects recruited had dyslipidemia in the form of elevated triglycerides (> 150 mg/dL) or cholesterol (>200 mg/dL) or low HDL (<50 mg/dL). Subjects were excluded if they were on medications for any chronic disease (cancer, cardiovascular disease, diabetes mellitus), pregnant or lactating, used any form of tobacco products, consumed alcohol (>1 oz/day), used mega doses of antioxidants or fish oil supplements (> 1 g/day), or had any abnormalities in hematology, liver, renal, and thyroid function tests which were confirmed with screening laboratory reports. Subjects were recruited through flyers and e-mail advertisements and were scheduled for a screening blood draw following an initial telephone screen. The 4-week study was conducted according to the guidelines laid down in the Declaration of Helsinki and all procedures involving human subjects were approved by the Institutional Review Board at Oklahoma State University. Written informed consent was obtained from each subject.

Subjects who qualified were asked to refrain from other sources of berries, green tea, cocoa, and soy products while on the study. These were the commonly consumed flavonoid-rich foods by the enrolled subjects as identified by a screening food frequency questionnaire specific for flavonoids. Subjects were asked to consume 2 cups of strawberry drink per day; each cup had 25 g of freeze-dried strawberry powder, one cup of water, one teaspoon of artificial sweetener Splenda^®^, and one teaspoon vanilla essence. The freeze-dried strawberry powder was donated by the California Strawberry Commission (Watsonville, CA, USA). All participants made 3 day (Monday, Wednesday, and Friday) per week visits to drink the first cup in the morning under observation by the research staff and were provided with the remaining supply of the strawberry drink in containers. Subjects were asked to drink the second cup at least six hours later in the day, and were instructed to keep the drink under refrigeration, avoid exposing the drink to direct heat or light or avoid consuming the strawberry drink with any other snack, lunch or dinner. Subjects were asked to bring back any unconsumed or left-over drink to assess unmonitored compliance. The nutritional value of the freeze-dried strawberry powder has been illustrated in Table 1. The freeze-dried strawberry powder was approximately 10% of the fresh weight of strawberries and had no added ingredients in the powder.

**Table 1:** Composition of freeze-dried strawberry powder. Values per 50 g of the freeze-dried strawberry powder (10% fresh weight)

Carbohydrates (g)	33.0
Protein (g)	3.5
Fat (g)	0.5
Calories (kcal)	150.0
Moisture (%)	10.0
Ash (g)	3.17
Vitamin C (mg)	109.0
Total Phenolics (mg)^1^	2006.0
Total Anthocyanins (mg)^2^	154.0
Ellagic acid (mg)	41.0
Phytosterols (mg)	50.0
Total dietary fiber (%)	8.0

Source: California Strawberry Commission (Watsonville, CA, USA)

^1^expresssed as mg gallic acid equivalents

^2^expressed as mg cyanidin-3-glucoside equivalents

Body weight, height, blood pressure, waist circumference measurements and fasting blood draws by a certified phlebotomist were conducted on the screening visit (baseline) and at final visit at the end of four weeks of the study. Systolic and diastolic blood pressure was measured in mm Hg using Spot Vital Signs Device (Welch Allyn, Skaneateles Falls, NY). Participants were asked to lie down and relax for approximately 8-10 minutes, following which three blood pressure measurements were recorded at an interval of 5 minutes at screen and 4 weeks of the study. Subjects were asked to maintain their usual diet, physical activity and lifestyle while enrolled in the study. Subjects were compensated on a weekly basis.

### Blood collection and analyses

Fasting blood samples (45 mL) were collected in SST tubes and in tubes containing EDTA as the anticoagulant at screen (baseline) and four weeks of the study following a 12-hour fast by the subjects. Serum and plasma were separated by centrifugation at 3000 rpm for 10 minutes at 4°C using Centrifuge 5810 R (Eppendorf, Hamburg, Germany). Serum and EDTA-plasma samples were sent to Stillwater Medical Center for analyses of serum glucose, lipids, albumin, liver, renal, and thyroid functions tests, and hematocrit and hemoglobin assays using standard laboratory techniques. Plasma and serum samples were stored at -80°C for subsequent analyses of biomarkers of oxidative stress and inflammation, and plasma ellagic acid.

### Dietary analyses

Dietary analyses were conducted at baseline and four weeks of the study. Subjects were asked to maintain a detailed 3-day food records which were analyzed by a trained registered dietitian using Food Processor (version 8.3, ESHA Research Inc.). Subjects were also asked to provide food labels or recipes for accurate analyses of their intakes.

### Biomarkers of oxidative stress and inflammation

Plasma concentrations of ox-LDL were measured in duplicate with ELISA kits (Mercodia, Uppsala, Sweden) according to the manufacturer's instructions. Lipid peroxidation was measured in serum as malondialdehyde (MDA) and 4-hydroxynonenal (HNE), using a colorimetric assay according to the manufacturer's protocol (LPO-586™, Oxis Health Products, Inc., Portland, OR). The average intra-assay CV for ox-LDL and MDA & HNE were 5.2 and 3.56%, respectively. Plasma hs-C-reactive protein (CRP) was measured using a quantitative sandwich enzyme immunoassay technique (R&D Systems, Minneapolis, MN). Plasma adiponectin was measured using a Mercodia Adiponectin ELISA, a solid phase two-site enzyme immunoassay (Mercodia, Uppsala, Sweden). The average intra-assay CV for hsCRP and adiponectin were 3.5 and 4.8%, respectively. Plasma ellagic acid was measured using HPLC-UV procedures as described previously by Seeram et al. [18]. The minimum detectable level of ellagic acid in our assay was 3.2 ng/mL. The inter-assay CV was 7.8%.

### Statistical analyses

Paired *t *tests were used for continuous variables, which had an overall normal distribution. Data were graphed for outliers. Variables were compared at baseline and at four weeks of the study using SPSS^® ^16.0 for Windows (SPSS Inc., Chicago, IL, USA). Our study was adequately powered to assess differences in the variables of interests with a sample size of sixteen. Statistical significance was accepted at a probability value of < 0.05 (two-sided test).

## Results

Sixteen females completed the study. No significant outliers were detected. The study subjects were free from chronic disease and were not on any prescription medications on a regular basis. Nine out of sixteen subjects (56%) were taking a multivitamin/mineral supplement on a regular basis and continued to do so while in the study. No differences were found between the supplement users and non users on the parameters of interest. All subjects maintained their usual diet and exercise patterns during the 4-week study.

Serum total and LDL-cholesterol levels were significantly reduced at 4 weeks versus baseline as shown in Table 2. No significant differences were noted in case of fasting glucose, triglycerides, HDL- and VLDL-cholesterol levels. Body weight, waist circumference, systolic and diastolic blood pressure were not significantly affected by freeze-dried strawberry powder supplementation.

**Table 2:** Effects of FSP supplementation on anthropometrics, blood pressure, clinical variables, oxidative stress, and inflammation (Mean values and standard error).

**Variables**	**Baseline**		**Post-intervention (4 weeks)**	
	**(n 16)**		**(n 16)**	
	**Mean**	**SE**	**Mean**	**SE**

Body weight (kg)	102.0	5.6	102.6	5.7
BMI	38.6	2.3	38.78	2.3
Waist circumference (inches)	43.0	1.5	44.0	1.5
Systolic blood pressure (mm Hg)	135.0	3.3	134.5	3.5
Diastolic blood pressure (mm Hg)	88.0	2.6	87.5	2.5
Glucose (mmol/L)	5.22	0.2	5.18	0.1
Total cholesterol (mmol/L)	5.32	0.2	5.05	0.16*
Triglycerides (mmol/L)	1.7	0.16	1.8	0.18
LDL-cholesterol (mmol/L)	3.2	0.17	3.0	0.12*
HDL-cholesterol (mmol/L)	1.24	0.05	1.22	0.04
VLDL-cholesterol (mmol/L)	1.0	0.2	0.83	0.08
Oxidized LDL (U/L)	118.0	7.5	109.0	7.3
MDA & HNE (μM)	1.4	0.04	1.2	0.03*
				
hsCRP (mg/L)	9.5	0.5	9.3	0.5
				
Adiponectin (μg/mL)	9.7	1.0	11.2	1.3

FSP-freeze-dried strawberry powder; *significantly different from baseline (p < 0.05)

A decreasing trend in oxidized-LDL levels was observed. Oxidized-LDL decreased from 117.98 ± 7.51 U/l at baseline to 108.96 ± 7.27 U/l at four weeks (p = 0.123). Serum MDA & HNE showed a significant decrease at 4 weeks compared to baseline (p < 0.01). No significant differences were noted in CRP and adiponectin concentrations (Table 2). Based on our lower limit of detection for the assay (3.2 ng/mL), plasma ellagic acid was non-detectable in all subjects at baseline, and 13 out of 16 subjects showed an increase at 4 weeks. The average plasma level of ellagic acid at 4 weeks was 8.5 ± 4.3 ng/mL. However, no significant correlations were found between increased ellagic acid and other variables of interests.

As illustrated in Table 3, no significant differences were noted in dietary intakes at four weeks versus baseline.

**Table 3:** Dietary nutrient intakes before and after FSP supplementation (Mean values and standard error).

**Variables**	**Baseline**		**Post-intervention (4-weeks)**	
	**(n 16)**		**(n 16)**	
	**Mean**	**SE**	**Mean**	**SE**

Energy (kcal)	1738.28	103.3	1790.27	136.4
Protein (g)	67.89	5.68	76.05	6.05
Carbohydrate (g)	206.84	16.55	203.80	17.0
Fiber (g)	14.25	1.34	14.78	1.60
Total fat (g)	69.62	4.7	74.96	7.4
Saturated fat (g)	23.96	1.99	26.48	2.36
Monounsaturated fat (g)	15.92	2.15	18.99	2.84
Polyunsaturated fat (g)	7.12	1.01	9.17	2.0
Cholesterol (mg)	221.04	35.0	261.24	30.68
Carotenoids (RE)	258.35	75.2	291.15	54.0
Vitamin C (mg)	68.72	22.1	69.57	14.0
Vitamin E (mg)	3.68	0.73	4.58	1.13
				
Copper (mg)	0.63	0.1	0.76	0.1
Iron (mg)	12.14	1.25	13.23	1.2
Zinc (mg)	7.06	1.11	9.88	1.43

FSP-freeze-dried strawberry powder

The study had no drop-outs although two subjects complained of a feeling of tingling and numbness of the extremities and flatulence during the first few days of the study. However, these symptoms were temporary and did not affect the subjects' compliance in the study. The strawberry drink was well tolerated and all participants showed active compliance with the study schedule.

## Discussion

With the rising costs of health care and pharmacological interventions, the role of natural dietary measures in the prevention and treatment of cardiovascular diseases has gained special attention. To our knowledge this is the first study investigating the effects of freeze-dried strawberry powder supplementation on selected cardiovascular risk factors in women with metabolic syndrome. Serum cholesterol levels were lowered following four weeks of freeze-dried strawberry consumption, which suggests a need for further study to determine if this might be a potential dietary approach to lowering cholesterol levels in obese women. In our study, subjects consumed a daily dose of 50 g freeze-dried strawberry powder which is equivalent to approximately 500 g fresh strawberries (3.5 cups). Our study findings add to the scientific basis supporting the cardio protective role of fruits and vegetables in human diet [7,19]. Some of the known cardio protective agents in strawberries include phytochemicals, vitamin C, folic acid, potassium, fiber and phytosterols, contributing to the antioxidant, anti-inflammatory, and hypocholesterolemic effects of strawberries [1,15,20].

To our knowledge, the effects of strawberry consumption on features of metabolic syndrome, oxidative stress, and inflammation have not been investigated in obese adults. Jenkins et al. have previously reported the cardiovascular health benefits of strawberries [21]. In their study, 28 hyperlipidemic subjects with an average baseline total and LDL-cholesterol of 5.62 ± 0.14 mmol/L and 3.61 ± 0.13 mmol/L, respectively, did not show any significant differences in lipid levels following intake of 454 g fresh strawberries everyday for four weeks. However, the study showed a significant reduction in thiobarbituric acid-reactive substances in LDL, indicating reduced oxidative damage to lipids. It should be noted that subjects in their study were following a cholesterol-lowering diet including soy, viscous fiber, plant sterols, and nuts for a mean of 2.5 years prior to strawberry supplementation for 4 weeks. Thus, the antioxidant effects of strawberries could have been confounded by the various dietary modifications made by these subjects [22]. In our study, subjects had a lower baseline total and LDL-cholesterol levels (5.32 ± 0.2 mmol/L and 3.2 ± 0.17 mmol/L, respectively) compared to the reported data by Jenkins et al. which indicates that freeze-dried strawberry powder supplementation may reduce cholesterol in subjects with mild elevation than those with high cholesterol levels. Cholesterol-lowering effects of freeze-dried strawberry powder may be attributable to the phytosterol, fiber, or phytochemical content of strawberries. Phytosterols have been shown to inhibit cholesterol absorption and lower cholesterol levels in clinical studies [22,23]. Since plant foods are natural sources of phytosterols, supplementation of concentrated fruit powder may be a novel approach to lowering selected CVD risk factors in women with clinically significant obesity (BMI >35), as noted in our study. Furthermore, the total dietary fiber content of the strawberry drink (8 g/day) may also contribute to the cholesterol lowering effects. The decreasing trend in oxidized-LDL in our study bears consideration and further research. Because oxidized-LDL plays a critical role in the initiation and progression of atherosclerosis [24], long-term supplementation of antioxidant-rich fruits, like strawberries may be beneficial in slowing or reversing the process of atherosclerotic cardiovascular disease in obese women with metabolic risk factors.

Our study findings of a significant decrease in MDA & HNE support similar effects reported by Pajk et al. in a porcine model of oxidative stress following supplementation of a fruit mixture including strawberries [25]. Thus, further study is warranted to examine whether free radical damage can be significantly decreased by dietary intervention with freeze-dried strawberry powder. Due to the lack of a control group and the small sample size comprising only women, our study results cannot be widely generalized. But, the high compliance rate, bioavailability of strawberry ellagic acid, and tolerability of the freeze-dried strawberry drink, suggests that larger, controlled interventions using strawberry supplementation are feasible. Furthermore, since none of our subjects were on lipid-lowering medications, and no significant dietary changes were noted before and after supplementation, our data indicate that the improvements in lipids and lipid peroxidation may possibly be due to strawberry powder supplementation per se. Sub-analyses of the dietary data, among daily users of dietary supplements, did not show any significant differences at 4 weeks on variables of interests, suggesting that diet and supplementation did not confound our study findings. However, such effects need to be confirmed in well-controlled clinical trials.

Data from the National Health and Nutrition Examination Survey (2001-2002), reveal that US adults consume 53.5% whole fruit recommendations, mainly as apples, pears and bananas, while vegetable intake is significantly below the recommendations by the Dietary Guidelines for Americans, 2005. Fruit servings include grain-based desserts and fruit juices which are popular food items, but constitute a high-fat and high-sugar means of consuming fruits [26]. Thus, to increase the intake of antioxidant-rich fruits and vegetables in the diet, concentrated fruit powders blended as smoothies may be a more attractive and convenient approach, especially for adults who do not consume the recommended amounts of fruits and vegetables. Our data suggest the need for a randomized controlled trial including a well-defined population of adequate sample size in assessing the effects of freeze-dried berry powder, as antioxidants and anti-inflammatory agents in adults with obesity and related metabolic disorders.

## Conclusion

Freeze-dried strawberry powder (FSP), a concentrated source of strawberry polyphenolic flavonoids, fiber, and phytosterols is a novel dietary fruit supplement marketed by selected fruit growers and special promotion groups. Not enough scientific data is available on the health benefits of this product. Our study shows the potential role of FSP in lowering total and LDL-cholesterol, and lipid peroxidation in women with metabolic syndrome, and suggests the need for larger controlled trials.

## Competing interests

This study was supported by a grant from the College of Human Environmental Sciences at Oklahoma State University. Freeze-dried strawberry powder was kindly donated by the California Strawberry Commission. This study is part of our ongoing research on novel functional foods and CVD risk factors in humans.

## Authors' contributions

AB and NB conceived the idea for the study and implemented the study design. MW, KP, & BS administered the strawberry intervention, subject recruitment and follow-ups. BS also did blood draws at all visits. NB provided valuable statistical assistance. TL provided expertise on language and content. AB prepared the manuscript and all authors have read and approved the final manuscript.
